# ISarcoPRM algorithm for global operationalization of sarcopenia diagnosis

**DOI:** 10.18632/aging.206174

**Published:** 2024-12-11

**Authors:** Pelin Analay, Murat Kara, Levent Özçakar

**Affiliations:** 1 Department of Physical and Rehabilitation Medicine, Hacettepe University Medical School, Ankara, Turkey

**Keywords:** sarcopenia, ultrasound

We read with great interest the recently published article “Global consensus for sarcopenia” by Kirk et al. [1] We agree that an international consensus on sarcopenia diagnosis was essential for the use of common terminology in clinical practice and research. Further, we believe that this should be followed by the establishment of a globally standardized diagnostic algorithm. Herewith, we would like to underscore ISarcoPRM algorithm for the diagnostic evaluation of sarcopenia [2].

To begin, as demonstrated in GLIS consensus report, sarcopenia is an age-related disease and its definition includes loss of muscle mass and decline in muscle strength. [1] Age-related loss of muscle mass and strength develops primarily due to progressive loss of alpha motor neurons, resulting in denervation of muscle fibers that cannot be compensated by reinnervation with residual motor neurons. Since age-related alpha motor neuron loss cannot be prevented, loss of muscle fibers is inevitable. [3] However, compensation of muscle fibers’ denervation by residual motor neurons can be achieved through various interventions inducing neuronal and muscular plasticity, such as exercise, vitamin D, nutrition, [4] and management of obesity and related chronic metabolic disorders [5].

On the other hand, regarding the diagnosis of sarcopenia, one of the most important problems encountered is the use of different methods in detecting age-related loss of muscle mass. One of the most commonly used diagnostic methods, appendicular lean mass (ALM) measured by dual energy x-ray absorptiometry (DXA) has been reported to fail to precisely detect age-related loss of muscle mass [6] and its associations with sarcopenia-related outcomes (mortality, fractures, falls, disability) are found to be weak or inconsistent [7]. Further, since age-related loss of muscle mass begins primarily with the loss of type-2 (fast-twitch) muscle fibers, the loss of muscle mass is not homogeneous throughout the whole body [2, 6]. Therefore, ALM measurement should not be used in the diagnosis of sarcopenia. Instead, we advocate measuring quadriceps muscle mass, which is rich in type-2 muscle fibers. Similarly, as in the diagnosis of osteoporosis, we measure trabecular bone rich vertebrae for the diagnosis and the follow-up, we do not measure the total bone mass [6].

According to GLIS consensus report, muscle-specific strength (e.g. leg extension maximal strength standardized to quadriceps muscle volume) should be considered as part of the conceptual definition of sarcopenia. [1] If we measure the strength of a muscle for sarcopenia, why do we not use that muscle for the diagnosis of sarcopenia? Herein, we would like to underscore the ISarcoPRM - i.e. Sarcopenia Special Interest Group of ISPRM (International Society of Physical and Rehabilitation Medicine) - algorithm which is already being used for the diagnosis of sarcopenia [2]. In that algorithm, it is suggested to measure quadriceps muscle mass instead of ALM. Of note, quadriceps muscle mainly comprises type-2 (fast-twitch) fibers and is fundamental for mobility. Studies have shown that quadriceps muscle measurements had higher correlations with physical performance and strength tests when compared with the ALM measurements [2].

For the evaluation of quadriceps muscle mass, cross-sectional area or volume measurements may better be performed with magnetic resonance imaging or computed tomography. However, they are not as convenient as ultrasound (US) measurements in daily clinical practice where the patient might also be residing in nursing/intensive care. Additionally, there is simply no contraindication for the use of US in this group of elderly patients (commonly having metallic implants, hearing aids etc.) [6].

In conclusion, quadriceps muscle mass measurements, preferably by US and as recommended by the ISarcoPRM algorithm, holds great promise in the diagnosis of sarcopenia. Considering the consensus on the definition of sarcopenia in the GLIS report, measuring the quadriceps muscle mass (rather than ALM) is noteworthy/paramount for the assessment of muscle mass loss while diagnosing sarcopenia.
